# Consumption of minimally processed food is inversely associated with excess weight in adolescents living in an underdeveloped city

**DOI:** 10.1371/journal.pone.0188401

**Published:** 2017-11-30

**Authors:** Ingrid Sofia Vieira de Melo, Clara Andrezza Crisóstomo Bezerra Costa, João Victor Laurindo dos Santos, Aldenir Feitosa dos Santos, Telma Maria de Menezes Toledo Florêncio, Nassib Bezerra Bueno

**Affiliations:** 1 Departament of agroindustry, Federal Institute of Alagoas (IFAL), Murici, Alagoas, Brazil; 2 Faculty of Nutrition, Federal University of Alagoas (UFAL), Maceió, Alagoas, Brazil; 3 Universitary Center of Maceió (CESMAC), Maceió, Alagoas, Brazil; Temple University School of Medicine, UNITED STATES

## Abstract

**Background:**

The consumption of ultra-processed foods may be associated with the development of chronic diseases, both in adults and in children/adolescents. This consumption is growing worldwide, especially in low and middle-income countries. Nevertheless, its magnitude in small, poor cities from the countryside is not well characterized, especially in adolescents. This study aimed to assess the consumption of minimally processed, processed and ultra-processed foods by adolescents from a poor Brazilian city and to determine if it was associated with excess weight, high waist circumference and high blood pressure.

**Methods:**

Cross-sectional study, conducted at a public federal school that offers technical education together with high school, located in the city of Murici. Adolescents of both sexes and aged between 14–19 years old were included. Anthropometric characteristics (weight, height, waist circumference), blood pressure, and dietary intake data were assessed. Associations were calculated using Poisson regression models, adjusted by sex and age.

**Results:**

At total, 249 adolescents were included, being 55.8% girls, with a mean age of 16 years-old. The consumption of minimally processed foods was inversely associated with excess weight (Adjusted Prevalence Ratio: 0.61, 95% Confidence Interval: [0.39–0.96], P = 0.03). Although the consumption of ultra-processed foods was not associated with excess weight, high blood pressure and high waist circumference, 46.2% of the sample reported eating these products more than weekly.

**Conclusion:**

Consumption of minimally processed food is inversely associated with excess weight in adolescents. Investments in nutritional education aiming the prevention of chronic diseases associated with the consumption of these foods are necessary.

## Introduction

Currently there are different classification of foodstuffs based on the extent, nature and purpose of food processing [1, 2]. Monteiro et al. [2], proposed a division of foodstuffs into three groups. The first group classification, foods that are either fresh or minimally processed, includes unprocessed and minimally processed foods, whose the purpose is preserving them and the minimal processes are mostly physical; Processed foods, that its composition went through important modifications, such as the addition of substances, substances extracted and purified from unprocessed or minimally processed foods. In turn, ultra-processed foods are formulated almost entirely by processed ingredients and with a minimum of fresh ingredients and result from the processing of several foodstuffs, including ingredients from processed and minimally processed foods. This food products that are ready to eat or similar [2, 3]. The Food Guide for the Brazilian population is based in this classification proposed [4].

Because of its characteristics, such as high energy density, high content of fat and sugar and a strong presence of sodium, the consumption of these foods may be involved in the development of chronic diseases, both in adults and in children/adolescents [3, 5, 6]. There is some evidence linking the consumption of ultra-processed food to diseases, for example, Tavares et al. [7] showed that the prevalence of metabolic syndrome in a group of Brazilian adolescents was associated with the consumption of ultra-processed foods and Rauber et al. [8] showed that ultra-processed food consumption altered lipoprotein profiles in children. In addition, Louzada et al [9] demonstrated that the consumption of ultra-processed foods was positively associated with obesity in Brazilian adolescents and adults. Nevertheless, these studies did not specifically investigate the consumption of ultra-processed foods and its association with cardiovascular risk factors in low-income adolescents.

According to Stuckler et al. [10] the economic growth of a country seems to be closely related to the consumption of ultra-processed foods. However, currently, the growth rate of consumption of these foods is faster in low and middle-income countries, while in high-income countries it seems to be saturated. On the other hand, it is not surprising that urbanization is not the main contributing factor to the consumption of processed foods. This is probably because transnational corporations have invested severely over decades in the penetration of its products in rural areas [10].

The state of Alagoas is among the poorest of Brazil and Murici city is located in its countryside. According to official data of the Brazilian Institute of Geography and Statistics (IBGE, in Portuguese), there are about 26,710 inhabitants in Murici and the city’s Human Development Index was 0.527 in 2010, considered as low. In addition, the average monthly income of rural and urban households are about $265 and $321 dollars, respectively. The Gross Domestic Product of the city was about $ 2.037,00, in 2013 and 65% of the city’s households are beneficiaries of “Programa Bolsa Família”, a conditional cash transfer program from the federal government [11].

Therefore, the objective of the present study was to assess the consumption of minimally processed, processed and ultra-processed foods by adolescents from the *Instituto Federal de Alagoas* (IFAL)—Murici, and to determine if the intake was associated with the presence of excess weight, high waist circumference, and high blood pressure.

## Methods

This manuscript is reported according to the Strengthening the Reporting of Observational studies in Epidemiology (STROBE) guidelines.

### Ethical aspects

This study and the consent procedure were approved by the Ethics Committee in Research of the Centro Universitário de Maceió under protocol number 1588/12 in accordance with the principles expressed in the Declaration of Helsinki. All parents and/or legal responsible of the participants provided their written consent and all adolescents assented to participate.

### Sample and study design

This cross-sectional study included all students with regular attendance, of both sexes and aged between 14–19 years old, of the IFAL—Murici, Alagoas, Brazil. The adolescents were classified according to the parameters of the World Helth Organization (WHO) [12], which considers adolescents, young people in the age range of 10–19 years old. Pregnant or lactant girls were not included.

### Local

The state of Alagoas is among the poorest of Brazil and Murici city is located in the countryside of Alagoas. IFAL is a public federal school that offers technical education together with high school and it is located in 15 cities of Alagoas state, including Murici city.

### Anthropometric evaluation and blood pressure measurement

The measurement of anthropometric data followed the recommendations of Lohman et al. [13]. Body weight was measured using a calibrated portable digital scale, with a maximum capacity of 200 kg and 0.1-kg precision (110 CH; Welmy, São Paulo, Brazil). Height was measured using a wall stadiometer with a maximum capacity of 2,000 mm in 1-mm increments. The current nutritional status of the adolescents was obtained using the Z-score of the body mass index (BMI) for age, according to Onis et al.[14], where BMI-for-age greater than +1 standard deviation are classified as excess weight. Excess weight was considered a marker of cardiovascular risk, in the present study.

Measurements of waist circumference were collected using a tape measure with the adolescents standing still. Waist circumference was obtained at the midpoint of the distance between the last rib and the anterior superior iliac spine, according to WHO recommendations [15]. The cutoff points used to classify waist circumference were those suggested by Taylor et al. [16], which varies according to the age and sex of the adolescents. These cutoff points showed a high sensitivity and specificity to identify high trunk fat mass, a proxy of cardiovascular risk. Blood pressure was measured using calibrated monitors against mercury manometers (Model HEM-7113, Omron, Osaka, Japan). Three measurements for each adolescent, with intervals of five minutes between measurements, were conducted. High blood pressure, considered in the present study a surrogate marker of cardiovascular risk, was classified according to the American Academy of Pediatrics [17].

### Dietary intake data

Dietary intake was assessed using a validated Food Frequency Questionnaire (FFQ) [18], with slight modifications. This questionnaire asked the consumption frequency of 84 different foods. The consumption frequency ranged from less than one time per month (coded as 0) to more than one time per day (coded as 7). In addition, the 84 foods were divided into three subgroups: minimally processed foods, processed foods, and ultra-processed foods, according to the Brazilian Food Guide [4]. A trained individual applied the questionnaires and performed the coding and analysis of dietary assessments.

### Statistical analyses

Data are presented as absolute and relative frequencies. To assess the association between the consumption frequency of minimally processed, processed and ultra-processed foods and the variables that indicate cardiovascular risk factors, Poisson regression models with robust deviance estimation were built. In these models, prevalence ratios were calculated, adjusted for sex and age. To explore the association between ultra-processed food consumption and cardiovascular risk factors, multivariate Poisson regression analysis were conducted using as covariate each food one at a time, adjusting by age and sex, with the cardiovascular risk factors as outcomes.

## Results

At total, 249 adolescents were investigated. The characteristics of the sample and the most commonly reported foods are presented in Tables 1 and 2, respectively.

**Table 1:** Characteristics of the included adolescents (n = 249).

Characteristic	Frequency	%
Age		
14–16 years	144	57.8
17–18 years	105	42.2
Sex		
Male	110	44.2
Female	139	55.8
Height for age		
Normal (Z-score > -2)	242	97.2
Short stature (Z-score < -2)	7	2.8
Body mass index for age		
Underweight (Z-score < -2)	8	3.2
Normal (-2 < Z-score < +1)	181	72.7
Excess weight (Z-score > +1)	60	24.1
Waist Circumference		
Normal	211	84.7
Cardiovascular risk	38	15.3
Blood Pressure		
Normal	213	85.5
High	36	14.5
Intake of minimally processed foods		
Less than weekly (Median score < 3)	108	43.4
Weekly or more (Median score ≥ 3)	141	56.6
Intake of processed foods		
Less than weekly (Median score < 3)	154	61.8
Weekly or more (Median score ≥ 3)	95	38.1
Intake of ultra-processed foods		
Less than weekly (Median score < 3)	134	53.8
Weekly or more (Median score ≥ 3)	115	46.2

**Table 2:** Foods reported in the food frequency questionnaire.

Food	Median*	Interquartile Range
**Minimally processed**		
Milk	3,0	2,0
Coffee	4,0	6,0
Tea	0,0	3,0
Polished rice	5,0	1,0
Brown rice	0,0	1,0
Couscous, tapioca	4,0	2,0
Bean	5,0	1,0
Chickpeas/ soybeans	0,0	2,0
Egg	3,0	1,5
Bovine offal	0,0	2,0
Beef	3,0	4,0
Pork	1,0	3,0
Chicken offal	0,0	3,0
Chicken	3,0	2,0
Fish and sea food	2,0	3,0
Soup	2,0	2,0
Lettuce	3,0	5,0
Carrot	2,0	4,0
Cabbage, chard, cauliflower	0,0	3,0
Tomato	3,0	5,0
eggplant, beet, chayote, cucumber zucchini	0,0	3,0
Pumpkin	0,0	3,0
Orange	3,0	2,5
Banana	3,0	3,0
Apple, pear	3,0	3,5
Papaya	2,0	3,0
Melon, watermelon	2,0	2,0
Pineapple	2,0	3,0
Mango	2,0	2,0
Guava	3,0	2,0
Grape	2,0	2,0
Peanuts, nuts, walnuts	0,0	3,0
Natural juice	4,0	3,0
**Processed**		
Mozzarella, parmesan, provolone Cheese	3,0	2,0
Ricotta cheese	0,0	0,5
Integral bread	3,0	5,0
Butter/ margarine	5,0	3,0
Jelly	0,0	1,0
Jerked beef	3,0	3,0
Olive oil	0,0	3,0
Added-salt in salad	3,0	5,0
Added-sugar in meal	0,0	5,0
Added-sugar in drinks	3,0	5,0
**Ultra-processed**		
Yogurt with sugar	3,0	1,0
White Bread	5,0	3,0
Cookie	3,0	3,0
Wafer	3,0	4,0
Cake	3,0	2,0
Corn flakes	0,0	3,0
Cream cheese	2,0	3,0
Mayonnaise	1,0	3,0
Chocolate milk	3,0	3,0
French fries, fried cassava	3,0	1,0
Cassava flour	3,0	3,0
Hamburger in the meal	0,0	3,0
Hamburger at the lunch	2,0	3,0
Sausage in the meal	3,0	3,0
Sausage at the lunch	0,0	3,0
Pork sausage	0,0	3,0
Steak, sausage or chicken meatball	0,0	3,0
Ham	3,0	2,0
Mortadella	3,0	3,0
Salami	2,0	3,0
Turkey breast	0,0	2,0
Pasta, lasagne, gnocchi	3,0	1,5
Fried snacks	3,0	3,0
Roasted snacks	2,0	3,0
Pizza	2,0	2,0
Crackers	3,0	2,0
Popcorn	3,0	2,0
Industrialized juice	0,0	3,0
Instant juice	2,0	3,0
Soft drink	3,0	3,0
Candies, lollipops, chewing gum	3,0	3,5
Chantilly, milk cream, canned milk	2,0	3,0
Chocolate	3,0	1,0
Pies, puddings, mousses	2,0	3,0
Ice cream, milkshake	2,0	1,0

*Median calculated from the scale ranging (0—does not consume; 1 -consumed less than monthly; 2—consumed 1–3 times per month; 3—consumed 1–3 times in the week or 4–10 times per month; 4—consumed 4–6 times in the week; 5—one times a day or 7 times a week; 6—more than once a day)

The mean age of the sample was 16 years and 139 individuals (55.8%) were female. The prevalence of excess weight was 24.1%. Also, 15.3% and 14.5% of the adolescents showed cardiovascular risk according to waist circumference, and blood pressure classification, respectively. Although 46.2% of the adolescents reported more than weekly consumption of ultra-processed foods, there were no significant associations of these variables with the consumption of ultra-processed foods (Table 3). On the other hand, 56.6% of the adolescents reported more than weekly consumption of minimally processed foods, which was higher among eutrophic adolescents than in adolescents with excess weight (Adjusted Prevalence Ratio: 0.61, 95% Confidence Interval: [0.39–0.96], P = 0.03) (Table 3).

**Table 3:** Association between frequency of food intake (less than weekly or weekly or more) for each type (ultra-processed or minimally processed) and variables indicating cardiovascular risk (n = 249).

Food intake	Risk factor				PR^1^	95% CI	P-value
**Min. processed**	**BMI-for-age**						
	No excess weight (n = 189)		Excess weight (n = 60)				
	N	%	N	%			
Less than weekly	75	39.7	33	55.0	1	-	-
Weekly or more	114	60.3	27	45.0	0.61	0.39–0.96	0.03
	**Waist circumference**						
	Normal (n = 211)		High (n = 38)				
	N	%	N	%			
Less than weekly	90	42.7	18	47.4	1	-	-
Weekly or more	121	57.3	20	52.6	0.90	0.51–1.60	0.73
	**Blood pressure**						
	Normal (n = 213)		High (n = 36)				
	N	%	N	%			
Less than weekly	96	45.1	12	33.3	1	-	-
Weekly or more	117	54.9	24	66.7	1.67	0.89–3.15	0.11
**Processed**	**BMI-for-age**						
	No excess weight (n = 189)		Excess weight (n = 60)				
	N	%	N	%			
Less than weekly	113	59.8	41	68.3	1	-	-
Weekly or more	76	40.2	19	31.7	0.74	0.46–1.21	0.24
	**Waist circumference**						
	Normal (n = 211)		High (n = 38)				
	N	%	N	%			
Less than weekly	135	64.0	19	50.0	1	-	-
Weekly or more	76	36.0	19	50.0	1.63	0.92–2.89	0.09
	**Blood pressure**						
	Normal (n = 213)		High (n = 36)				
	N	%	N	%			
Less than weekly	132	62.0	22	61.1	1		
Weekly or more	81	38.0	14	38.9	1.03	0.56–1.90	0.91
**Ultra-processed**	**BMI-for-age**						
	No excess weight (n = 189)		Excess weight (n = 60)				
	N	%	N	%			
Less than weekly	98	51.98	36	60.0	1	-	-
Weekly or more	91	48.1	24	40.0	0.76	0.47–1.22	0.25
	**Waist circumference**						
	Normal (n = 211)		High (n = 38)				
	N	%	N	%			
Less than weekly	112	53.1	22	57.9	1	-	-
Weekly or more	99	46.9	16	42.1	0.94	0.51–1.72	0.85
	**Blood pressure**						
	Normal (n = 213)		High (n = 36)				
	N	%	N	%			
Less than weekly	117	54.9	17	47.2	1	-	-
Weekly or more	96	45.1	19	52.1	1.55	0.83–2.91	0.16

^1^Prevalence ratio, calculated through a Poisson regression model adjusted by age and sex

When the association between ultra-processed foods consumption and cardiovascular risk factors were further explored, using each food one at a time in the regression models, sausage was the only food that the consumption was positively associated with any of the cardiovascular risk factor studied. Sausage consumption was positively associated with the prevalence of excess weight (Adjusted Prevalence Ratio: 1.13, 95% Confidence Interval: [1.01–1.26], P = 0.03).

## Discussion

The consumption of traditional foods apparently still seems to be a positive aspect in the studied adolescents, consistent with the poor, small town characteristics of where they live. The study by Louzada et al. [9], who studied the consumption of ultra-processed foods and obesity in Brazilian adolescents and adults, found that the amount of consumption (in % of total energy intake) of minimally processed foods was 68.6%, twice that of ultra-processed foods, in individuals older than 10 years of age. The consumption of minimally processed foods was inversely associated with overweight in the studied adolescents. A similar study showed that the consumption of processed foods was inversely associated with weight gain [19].

The consumption of ultra-processed foods was not associated with excess weight and cardiovascular risk markers in the present study. Nevertheless, sausage consumption was positively associated with excess weight. The classification of foods proposed by the Food Guide for the Brazilian Population [4], which was used in the present study, includes a wide range of foods in the ultra-processed category. For example, foods such as white bread and yogurt with sugar are included in the same category of soft drinks, confectionery and sausage. Hence, it is possible that due to this variety of foods included in the ultra-processed category, we were not able to find significant associations between the whole category and the cardiovascular risk factors. However, when sausage, a food which processing level is notably high, the association was found.

Although the consumption of ultra-processed foods was not associated with excess weight and cardiovascular risk markers in this study, it was high among all adolescents, apparently overcoming the financial constraints of a very poor city. It denotes the need of investments in nutritional education aiming the prevention of chronic diseases associated with the consumption of these foods. It also highlights the need to strengthen the traditional eating habits of this population. The present study has some limitations. First, the semi-quantitative FFQ was used to assess dietary intake, which prevents from undertaking more accurate analyses. Nevertheless, it was used a validated FFQ that was applied by a single trained individual, which decrease the bias potential of the data. Second, no physical activity data was assessed in the present sample. Since it is usual that low-income communities present high exercise physical activity levels, this factor might have attenuated the association between consumption of ultra-processed foods and cardiovascular risk factors.

In conclusion, the consumption of minimally processed food is inversely associated with excess weight in adolescents living in an underdeveloped city. Investments in nutritional education aiming the prevention of chronic diseases associated with the consumption of these foods are necessary.

## Supporting information

S1 File Data set. (XLSX)
